# Leukemia Stem Cell Release From the Stem Cell Niche to Treat Acute Myeloid Leukemia

**DOI:** 10.3389/fcell.2020.00607

**Published:** 2020-07-09

**Authors:** Alicia Villatoro, Joanna Konieczny, Vincent Cuminetti, Lorena Arranz

**Affiliations:** ^1^Stem Cell Aging and Cancer Research Group, Department of Medical Biology, Faculty of Health Sciences, UiT – The Arctic University of Norway, Tromsø, Norway; ^2^Norwegian Center for Molecular Medicine (NCMM), University of Oslo, Oslo, Norway

**Keywords:** leukemia stem cell, hematopoietic stem cell, homing, mobilization, adhesion, hematopoietic stem cell niche, acute myeloid leukemia, clinical trials

## Abstract

Acute myeloid leukemia (AML) is a heterogeneous, complex, and deadly disease, whose treatment has hardly evolved for decades and grounds on the use of intensive chemotherapy regimens. Chemotherapy helps reduce AML bulk, but promotes relapse in the long-run by selection of chemoresistant leukemia stem cells (LSC). These may diversify and result in progression to more aggressive forms of AML. *In vivo* models suggest that the bone marrow stem cell niche helps LSC stay dormant and protected from chemotherapy. Here, we summarize relevant changes in stem cell niche homing and adhesion of AML LSC vs. healthy hematopoietic stem cells, and provide an overview of clinical trials aiming at targeting these processes for AML treatment and future directions within this field. Promising results with various non-mutation-targeted novel therapies directed to LSC eradication via interference with their anchoring to the stem cell niche have encouraged on-going or future advanced phase III clinical trials. In the coming years, we may see a shift in the focus of AML treatment to LSC-directed therapies if the prospect of improved cure rates holds true. In the future, AML treatment should lean toward personalized therapies using combinations of these compounds plus mutation-targeted agents and/or targeted delivery of chemotherapy, aiming at LSC eradication with reduced side effects.

## Introduction: Acute Myeloid Leukemia

Acute myeloid leukemia (AML) is a heterogeneous disease characterized by aberrant myeloid lineage proliferation and differentiation, accompanied by at least one clonal somatic abnormality on mutational profiling in more than 97% of patients (Coombs et al., 2016). AML is the most common acute leukemia in adults, with an average age at diagnosis of about 68 years. The American Cancer Society’s estimates for AML in the United States for the year 2020 are about 19,940 new cases and 11,180 deaths. Most of the patients die from the disease. The standard treatment for AML has changed little for decades, and consists of induction chemotherapy with cytarabine and anthracycline, followed by either consolidation chemotherapy or allogeneic stem cell transplantation, depending on factors like age. The 5-year survival is poor due to relapse in more than 70% of patients (Burnett et al., 2011). In many instances, relapses are caused by treatment-selection of chemoresistant leukemic cells, able to sustain leukemia and give rise to more differentiated cells, i.e., leukemia stem cells (LSC). These are a rare cell population with main hematopoietic stem cell (HSC) features, such as quiescence or dormancy and self-renewal (Buzzai and Licht, 2008; Meacham and Morrison, 2013), still different from normal HSC and other AML progenitors. AML LSC not only may persist after chemotherapy, but may also diversify clonally and drive progression to more aggressive forms of the disease, leading to the fatal outcomes (Vetrie et al., 2020). With these precedents, any potential therapeutic strategy for AML should ultimately aim at their eradication.

## Leukemia Stem Cells

Initially, LSC were characterized immunophenotypically as CD34^+^ CD38^–^ cells in peripheral blood of AML patients, and functionally capable of initiating AML after transplantation in single cell dilution in immunocompromised NOD/SCID IL2Rγ^–/–^ (NSG) mice (Lapidot et al., 1994). Both LSC and normal residual HSC are enriched within the CD34^+^ CD38^–^ fraction in AML patients, which generated the need to identify additional markers to distinguish both. Currently, CD34^+^ CD38^–^ CD123^+^ immunophenotyping is highly standardized to quantify LSC, and seems to allow this distinction (Jordan et al., 2000). Expression of the interleukin-3 receptor alpha chain, CD123, in CD34^+^ CD38^low/–^ AML blasts, is a prognostic factor for poor patient outcome (Vergez et al., 2011), and neutralizing antibody reduced AML burden and impaired secondary transplantation in NOD/SCID mice (Jin et al., 2009). An additional marker to discriminate LSC and HSC is the T-cell immunoglobulin mucin-3 (TIM-3) (Jan et al., 2011), whose targeting impaired LSC but not HSC reconstitution (Kikushige et al., 2010). CD96 belongs to the immunoglobulin superfamily and it is also enriched in CD34^+^ CD38^–^ AML blasts vs. normal CD34^+^ CD38^–^ CD90^+^ HSC (Hosen et al., 2007). Only CD96^+^ AML cells showed engraftment capacity in the bone marrow of newborn Rag2^–/–^ IL2Rγ^–/–^ mice (Hosen et al., 2007). Additional surface markers reported as enriched in LSC vs. HSC are CD47, CD244, CD33, and C-type lectin-like molecule-1, among others (van Rhenen et al., 2007; Majeti et al., 2009; Krupka et al., 2014; Zhang et al., 2017). As of today though, no universal surface marker has been identified on CD34^+^ CD38^–^ LSC across AML patients that is absolutely not expressed on AML blasts or normal HSC.

Functional human LSC in the bone marrow of transplanted mice have also been found within cell fractions expressing lineage markers, CD38, or CD45RA (Sarry et al., 2011), suggesting that these cells are not restricted to the immature phenotype. Later, combined genetic and functional analysis of purified subpopulations and xenografts from paired diagnosis/relapse samples showed that relapse may originate from rare LSC or from larger subclones of immunophenotypically committed leukemia cells that retained stemness transcriptional signatures (Shlush et al., 2017). These findings captured the complexity of the cellular origins of relapse in AML, but also emphasized the importance of targeting stemness in AML treatment. In fact, a strong stemness signature in AML blasts predicts initial therapy resistance and poor outcomes with all available treatments, including allogeneic stem cell transplantation (Ng et al., 2016). Thus, when testing the efficiency of novel therapies, it is essential that eradication of LSC is evaluated functionally in xenografts by *in vivo* serial transplantations of at least secondary recipients that would confirm lost long-term engraftment, self-renewal and potential to regenerate AML.

Stemness is determined by both cell-intrinsic and cell-extrinsic cues, derived from the microenvironment or niche where the cell resides. Regarding cell-intrinsic features, advances in single-cell RNA and DNA analyses are allowing deeper understanding of clonal composition, evolution and hierarchy. Recent work combining both approaches confirmed primitive AML cells as prognostic hallmarks, and further showed co-expression of stemness and myeloid priming genes, and abundancy of prototypic genetic lesions like FLT3-ITD in these cells. Conversely, differentiated monocyte-like AML cells expressed immunomodulatory genes (van Galen et al., 2019). Targeted deep sequencing combined with single-cell sequencing revealed that stem cells in myelodysplastic syndromes (MDS) have high subclonal complexity, and different subclones contribute to generation of blasts or progression to AML in parallel. Subclones that expand during AML transformation are present but not detectable in MDS blasts, and targeting these clones in MDS patients will greatly reduce the proportion of secondary AML (Chen et al., 2019). Mutations associated with progression to AML included those in RUNX1, NRAS, ERG, ATRX, NTRK3, and DUSP22 (Chen et al., 2019). Ideally, evaluation of LSC should combine studies of *in vivo* function, with single-cell stemness signatures and mutational profiling.

**FIGURE 1:**
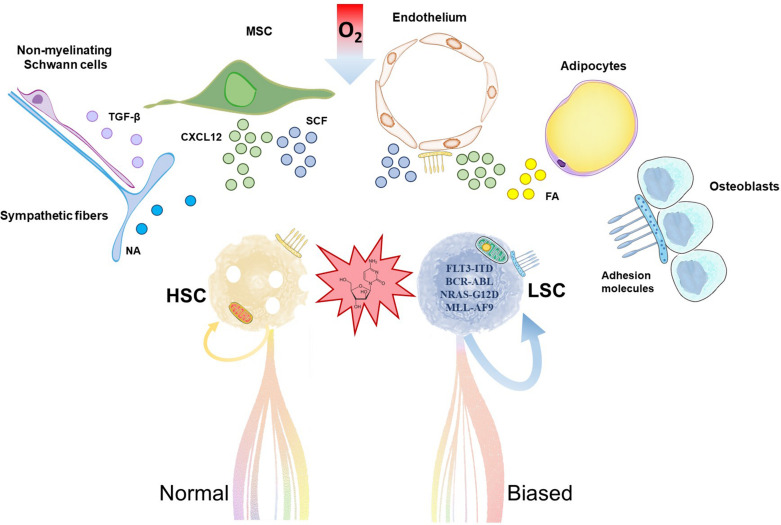
Hematopoietic stem cells (HSC) and leukemia stem cells (LSC) share common stem cell niches in the bone marrow. Accumulation of mutations and metabolic reprogramming in LSC lead to increased self-renewal and myeloid-biased aberrant differentiation in acute myeloid leukemia. Chemoresistant LSC outcompete HSC, trigger relapse and ultimately fatal patient outcome. HSC and LSC reside in stem cell niches formed by cells of hematopoietic and non-hematopoietic or stromal origin. Relevant stromal components are depicted in the illustration. These cells support HSC/LSC maintenance by provision of soluble factors, cell-cell interactions, extracellular matrix and metabolic signals like hypoxia, among others. MSC, mesenchymal stromal cell; NA, noradrenaline; TGF-β, transforming growth factor-beta; CXCL12; C-X-C motif chemokine 12; SCF, stem cell factor; FA, fatty acids.

Stem cell ability to self-renew and retain its identity depends on the microenvironment provided by non-HSC cells in the neighborhood, which includes cell-to-cell interactions, secreted factors, inflammation, extracellular matrix, and metabolic signals such as hypoxia, among others (Lane et al., 2014; Figure 1). HSC reside in hypoxic environments in the bone marrow and preferentially use glycolysis to obtain energy, which supports long-term self-renewal and quiescence (Suda et al., 2011; Arranz et al., 2013). LSC share metabolic features that include low mitochondrial activity, which limits the novel therapies that can be developed, but they are particularly dependent on low but intact mitochondrial oxidative phosphorylation (Lagadinou et al., 2013). Oxidative phosphorylation in chemotherapy-resistant leukemia cells is fueled by mitochondrial fatty acid oxidation (Farge et al., 2017). Several publications show promising therapeutic value for inhibition of fatty acid oxidation in mouse models of human AML (Cuminetti and Arranz, 2019), but this strategy may have a negative impact on HSC maintenance too (Ito et al., 2012).

The HSC niche in the bone marrow is composed of different cell populations of both hematopoietic and non-hematopoietic or stromal origin, including osteoblasts, endothelial cells, mesenchymal stromal cells (MSC), adipocytes, non-myelinated Schwann cells and sympathetic neurons (Sanchez-Aguilera and Mendez-Ferrer, 2017; Figure 1). Recent body of evidence suggests that the HSC niche is altered by AML. This perturbs healthy HSC and contributes to the competitive advantage of LSC, as these cells seem to have different degree of dependency and sensitivity to survival, anchoring or regulatory signals from the HSC niche. For example, adipocytes are important components of the HSC niche (Cuminetti and Arranz, 2019) whose role is disrupted in xenograft models of human AML (Boyd et al., 2017). *In vivo* administration of PPARγ agonists induced bone marrow adipogenesis, and rescued healthy hematopoiesis while suppressing AML in these models (Boyd et al., 2017). MSCs are major regulators of hematopoiesis by production of a variety of factors that regulate HSC quiescence and retention/homing, remarkably stem cell factor (SCF) and C-X-C motif chemokine 12 (CXCL12), respectively (Mendez-Ferrer et al., 2010; Ding et al., 2012; Kunisaki et al., 2013). In a syngeneic MLL-AF9 AML transplantation model, MSC acquired an osteoblastic profile and downregulated the expression of both factors (Hanoun et al., 2014). Previous work showed that, in xenografts, LSC home within the endosteal bone marrow, where they are protected from chemotherapy-induced apoptosis in a quiescent status (Ishikawa et al., 2007).

This variety of cell types and soluble and membrane factors coordinate their activities for a fine-tuned regulation of HSC location and function. Anchoring of HSC to the HSC niche is mediated by adhesion molecules, which maintain HSC attached to the cellular components of the HSC niche and to the matrix or release them when mobilization is required. For instance, CXCL12 activates the integrins lymphocyte function associated antigen-1 (LFA-1) and very late activation antigen-4 (VLA-4) on CD34^+^ cells to bind their respective ligands in the niche, and treatment of these cells with antibodies to VLA-4 or VLA-5 prevented engraftment in NSG mice (Peled et al., 2000). It is not surprising then that adhesion molecules regulate a variety of HSC functions beyond migration and homing, including proliferation, differentiation, apoptosis, and engraftment (Levesque and Winkler, 2016). In AML, LSC adhesion to the vascular niche protects them from chemotherapy (Winkler et al., 2014), whereas detachment allows colonization of secondary sites (Gruszka et al., 2019). In this scenario, the purpose of this review is (1) to summarize relevant changes in homing and adhesion in AML LSC, (2) to provide an overview of recent and current clinical trials aiming at targeting these processes for AML treatment, and (3) to provide the grounds for future directions within this field.

## Soluble Factors Anchoring LSC to the Stem Cell Niche and Their Clinical Application in AML

### CXCL12

**FIGURE 2:**
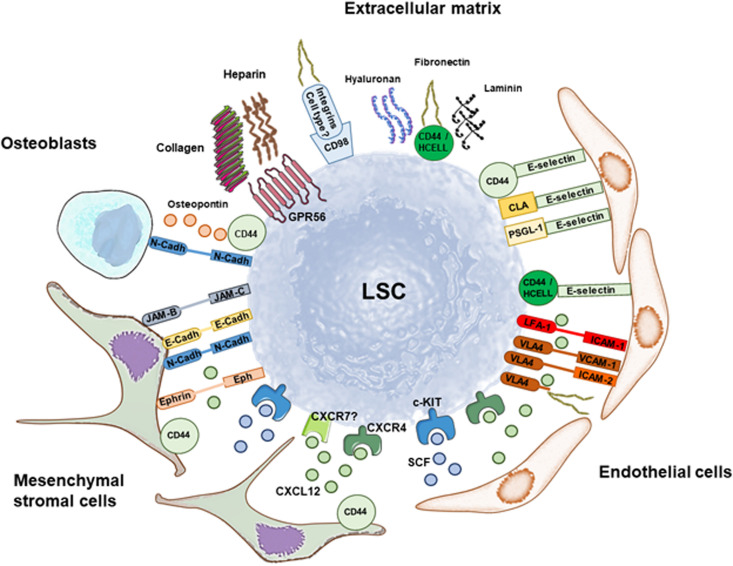
Retention of leukemia stem cells (LSC) in the stem cell niche through soluble factors and multiple adhesion molecules. Summary of interactions involved in LSC retention that are discussed in this Review. The main soluble factor that mediates maintenance of LSC in the stem cell niche is CXCL12 binding to CXCR4 (and maybe CXCR7), whereas SCF binding to c-KIT promotes quiescence and self-renewal. Mesenchymal stromal cells and endothelial cells are great producers of both soluble factors. LSC anchoring to the stem cell niche is additionally mediated by a variety of adhesion molecules that maintain cell-cell interactions with mesenchymal stromal cells, endothelial cells and osteoblasts, and interactions with the matrix. Preclinical and clinical data show that targeting LSC anchoring to the stem cell niche through disruption of these pathways has therapeutic potential against AML.

HSC and LSC homing and retention are regulated through interaction of CXCL12 to its receptor, C-X-C chemokine receptor type 4 (CXCR4) (Figure 2). CXCR4 expression levels in CD34^+^ leukemia cells are associated with poor survival and higher probability of relapse in AML patients (Rombouts et al., 2004; Spoo et al., 2006). In fact, chemotherapy may induce CXCR4 upregulation in some AML cell lines and patient samples, which results in chemotaxis and stromal protection from additional chemotherapy-induced apoptosis (Sison et al., 2013).

In contrast, in preclinical models of MLL-AF9^+^ AML, bone marrow MSC downregulate *Cxcl12* expression together with a variety of additional factors that support healthy hematopoiesis (Hanoun et al., 2014; Baryawno et al., 2019). This suggests that LSC outcompete HSC for the same niches, and transform them to disadvantage HSC in a self-maintenance cycle. HSC cycle actively and reduce their numbers after conditional deletion of CXCR4 in bone marrow cells, indicating that HSC maintenance relies on intact CXCL12/CXCR4 axis (Sugiyama et al., 2006). Taken together, if this is true for LSC, releasing them from the niche by targeting CXCL12/CXCR4 will force them into cycle and make them more sensitive to chemotherapy. A variety of CXCR4 inhibitors are currently in clinical trials pursuing this scientific paradigm. Some of them proved successful mobilizing leukemic cells or more specifically LSC, and sensitizing them to chemotherapy.

The small molecule CXCR4 antagonist plerixafor (AMD3100) is approved by the US Food and Drug Administration (FDA) in combination with granulocyte-colony stimulating factor (G-CSF) for mobilizing HSC to the bloodstream. In mouse models, administration of AMD3100 mobilized HSC and AML blasts with peak 3 h after injection, up to 25-fold and 9-fold, respectively, with the majority of the blasts being differentiated cells (Nervi et al., 2009). Treatment of AML mice with cytarabine plus AMD3100 decreased tumor burden and improved survival compared with chemotherapy alone (Nervi et al., 2009). In NOD/SCID mice, CXCR4 inhibitor AMD3465 mobilized human FLT3-ITD AML LSC, defined immunophenotypically as CD34^+^ CD123^+^, up to 7.5-fold (Zeng et al., 2009). In fact, when high levels of CXCR4 are expressed at the membrane of AML cells, blocking the receptor function with AMD3100 or the peptide TN140 was sufficient to reduce the number of LSC in NOD/Shi-SCID/IL2Rγ^–/–^ (NOG) mice (Zhang et al., 2012b). This was demonstrated by the reduced percentage of circulating human cells and higher survival in secondary recipient mice transplanted with cells sorted from TN140- or AMD3100-treated mice than in control mice (Zhang et al., 2012b).

In newly diagnosed AML patients, a phase I clinical study (NCT00990054) combined plerixafor with cytarabine and daunorubicin to determine the safety and tolerability of plerixafor. The toxicity of chemotherapy was not affected by plerixafor, and 67% of participants achieved complete remission (CR) (Uy et al., 2011). Table 1 shows an overview of the clinical trials potentially targeting LSC release from the stem cell niche to treat AML patients. In elderly patients, plerixafor was tested for its potential to induce sensitization of LSC to decitabine (NCT01352650). Plerixafor triggered LSC mobilization, however, these cells persisted in the bone marrow during treatment, and patients with more than 2-fold increase in LSC at cycle 3 relative to the previous study point relapsed before completing 7 cycles of therapy (Roboz et al., 2018). Overall response rate to this treatment was 43% and overall survival was 11 months, similar to previous reports of decitabine, which makes its benefit uncertain (Roboz et al., 2018). In contrast, a phase I/II clinical study (NCT00512252) used plerixafor to treat relapsed or refractory (R/R) AML patients in combination with mitoxantrone, etoposide, and cytarabine (MEC). Patients experienced a modest 2.5-fold mobilization of AML blasts with no preferential mobilization over healthy cells, and 39% achieved CR (Uy et al., 2012). This seems favorable compared with the 21% CR of MEC alone (Greenberg et al., 2004).

**TABLE 1:** Clinical trials targeting LSC release from the stem cell niche to treat AML patients.

Target	Drug	Mechanism	Combination therapy	Trial ID	Phase	No. evaluated patients	Disease	Age (years)	Efficacy			References
									%CR	%CR/CRi	OS (months)	
CXCL!2/ CXCR4 axis	Plerixafor; AMD3100	CXCR4 antagonist	Cytarabine and daunorubicin	NCT00990054	I	21	Newly diagnosed	57 (24–69)*	67	77	–	Uy et al., 2011
			
			Decitabine	NCT01352650	I	69	Newly diagnosed	73 (56–87)	35	42	11.2	Roboz et al., 2018
			
			Mitoxantrone, etoposide and cytarabine (MEC)	NCT00512252	I/II	46^Δ^	R/R	52 (18–70)*	39^Δ^	46^Δ^	8.2^Δ^	Uy et al., 2012
			
			G-CSF, fludarabine, cytarabine, and idarubicin	NCT01435343	I/II	41^Δ^	R/R	52 (18–64)^Δ^	44^Δ^	49^Δ^	9.9^Δ^	Martinez-Cuadron et al., 2018
			
			G-CSF, sorafenib	NCT00943943	I	21	R/R FLT3-ITD^+^	–	19	–	–	Andreeff et al., 2014
	
	Ulocuplumab; BMS-936564/MDX-1338	Anti-CXCR4 antibody	MEC	NCT01120457	I	43^Δ^	R/R	58 (21–79)*	–	51^Δ^	–	Becker et al., 2014
	
	BL-8040	CXCR4 antagonist peptide	Cytarabine	NCT01838395	IIa	16^Δ^	R/R	61 (43–74)*	–	38^Δ^	–	Borthakur et al., 2015
			
	LY2510924		Cytarabine and idarubicin	NCT02652871	I	11	R/R	55 (19–70)	18	36	7.8	Boddu et al., 2018
	
	CX-01; ODSH	Disrupts CXCR4/CXCL12, L- and P-selectin, ICAM-1, VCAM-1-mediated mechanism	Cytarabine and idarubicin	NCT02056782	I	12	Newly diagnosed	54 (22–74)	92	–	Not attained at 29.4	Kovacsovics et al., 2018
	
			Cytarabine and idarubicin	NCT02873338	II	66	Newly diagnosed	≥ 59*	–	89^Δ^	–	Kovacsovics et al., 2019

NF-κB pathway	Dexamethasone	Inhibitor of inflammatory cytokine secretion	Amsacrine-cytarabine or azacitidine	NCT03765541	III	142 estimated	R/R	≥ 18	–	–	–	Not yet recruiting
	
			Intensive chemotherapy	–	–	60	Treated patients, Hyperleukocytic AML	60 (18–75)	83^Δ^	–	39.8^Δ^	Bertoli et al., 2018

	Bortezomib	Proteasome inhibition	Tipifarnib	NCT00383474	I	26	R/R	70 (47–82)*	–	8	–	Lancet et al., 2011
	
			Idarubicin	NCT00382954	I	20	13 newly diagnosed,7 R/R	65 (40–83)	20	–	3.8	Howard et al., 2013
	
			Idarubicin and low-dose cytarabine	NCT00666588	II	14	R/R and secondary	10 (1.2–19.6)	21	50	∼12	Horton et al., 2014
							
			Etoposide and high-dose cytarabine			23^Δ^		6.1 (0.2–16.2)^Δ^	35^Δ^	39^Δ^	∼26	
	
	Ixazomib		MEC	NCT02070458	I	30	R/R	58 (31–70)	–	53	4.5	Advani et al., 2019
	
			No	NCT02030405	II	4	AML	≥ 18	0	0	–	Results Posted
	
	Clioquinol		No	NCT00963495	I	11	AML and others	≥ 18	–	–	–	No results Posted
	
Integrins / CD98	AS101	Disrupts VLA-4 function	Cytarabine and idarubicin; cytarabine	NCT01010373	II	12	AML, MDS	60–85	–	–	–	Suspended
	
	IGN523	Anti-CD98 monoclonal antibody	No	NCT02040506	I	19	R/R	71 (23–78)	0	0	–	Bixby et al., 2015
	
CD44 / E-Selectin	MLM-CAR44.1 T-cells	CD44v6 chimeric antigen receptor	Cyclophosphamide and fludarabine	NCT04097301	I/II	58 estimated	AML, multiple myeloma	1–75	–	–	–	Recruiting
	
	Uproleselan (GMI-1271)	E-selectin antagonist	MEC	NCT02306291	I/II	47^Δ^	R/R	59 (26–84)*	–	41^Δ^	8.8^Δ^	DeAngelo et al., 2018
					
			Cytarabine and idarubicin			25	Newly diagnosed	67 (60–79)	–	72	12.6	
	
			MEC or fludarabine, cytarabine and idarubicin (FAI)	NCT03616470	III	380 estimated	AML	18–75	–	–	–	Recruiting
	
			Cytarabine and daunorubicin; cytarabine	NCT03701308	II/III	670 estimated	AML	≥ 60	–	–	–	Recruiting
	
Eph	KB004	Recombinant antibody to EPHA3	No	NCT01211691	I/II	47	R/R	70 (25–89)	–	2	–	Swords et al., 2016

(-), data not available; (*), referred to total number of patients enrolled in the study; (△), referred to optimal doses; CR, complete remission; CRi, complete remission with incomplete blood count recovery; OS, overall survival; R/R, relapsed or refractory.

The anti-CXCR4 monoclonal antibody ulocuplumab (BMS-936564/MDX-1338) was also tested in combination with MEC in R/R AML patients (NCT01120457). Here, results showed 2- and 5-fold mobilization of leukocytes and AML blasts at day 8, respectively, and 51% of overall CR and CR with incomplete blood count recovery rate (CRi) (Becker et al., 2014). Interestingly, four patients had CR/CRi after a single dose of ulocuplumab monotherapy.

CXCR4 antagonist peptides seem to have more robust efficacy than plerixafor and ulocuplumab. BL-8040 is a short synthetic peptide with high affinity and long occupancy for CXCR4, which induces fast mobilization of CD34^+^ cells in healthy subjects (NCT02073019) (Abraham et al., 2017). Its efficacy was evaluated in a phase IIa study in R/R AML patients (NCT01838395). Strikingly, 2 days of BL-8040 monotherapy induced apoptosis and 40.2-fold mobilization of immature AML progenitors (CD45^+/low^ CD34^+^ CD117^+^ HLA-DR^+^), resulting in a median decrease of 57.7% in the number of bone marrow leukemia progenitor cells out of total CD45^+^ CD34^+^ normal progenitor cells. This reduction was accompanied by a 3.1-fold increase in granulocytes at day 3, further supporting a differentiation effect. In combination with cytarabine, a 38% CR/CRi was achieved (Borthakur et al., 2015), and overall survival of R/R AML patients significantly improved compared with historical data for cytarabine alone (Borthakur et al., 2018). Another CXCR4 antagonist peptide, LY2510924, was tested in R/R AML patients together with idarubicin and cytarabine (NCT02652871). Preliminary results also showed superior pharmacologic profile over plerixafor with high mobilization of leukocytes (6-fold), blasts (40-fold) and CD34^+^ cells (5-fold) at day 8 (Boddu et al., 2018). However, in this case the overall response rate of 36% of the combination was comparable with previous data on chemotherapy alone (Short et al., 2018).

Low anticoagulant 2-O, 3-O desulfated heparin (ODSH, CX-01) is a porcine intestinal heparin derivative that retains several heparin anti-inflammatory properties with reduced anticoagulation effect (Rao et al., 2010). Heparin bound to CXCL12 disrupting the interaction of the chemokine to heparan sulfate on cells (Sadir et al., 2001), so it is the chemokine that it is targeted by this strategy. Heparin blocked CXCL12- and also L-selectin- and P-selectin-mediated retention of cells in bone marrow (Zhang et al., 2012a). In addition, heparin reduced acute promyelocytic leukemia cell line NB4 adhesion to the immortalized human microvascular endothelial cell line-1 (HMEC-1), and the expression of intercellular adhesion molecule 1 (ICAM-1) and vascular cell adhesion molecule 1 (VCAM-1) on HMEC-1 cells (Vignoli et al., 2018). *In vivo*, disruption of CXCL12 interaction with their respective ligands LFA-1 and VLA-4 may further contribute to this effect. CX-01 was tested in a phase I pilot study in newly diagnosed patients with AML receiving cytarabine and idarubicin induction or cytarabine consolidation chemotherapy (NCT02056782). Preliminary results showed excellent morphologic CR achievement after 1 induction (92%), with median disease-free survival of 14.8 months, and median overall survival not attained at the maximum follow-up time (29.4 months) (Kovacsovics et al., 2018). Recently, a phase II study (NCT02873338) carried out by the same group in elderly patients (older than 59) confirmed a high 89% CR and CRi in patients treated with chemotherapy and high concentration of CX-01, compared to 58% in patients treated with chemotherapy and low CX-01 concentration or 50% in patients that received chemotherapy alone (Kovacsovics et al., 2019).

CXCL12 may alternatively bind to CXCR7 in several AML cell lines, whereas healthy CD34^+^ HSC were reported to express low levels of CXCR7 (Tarnowski et al., 2010). CXCR7 expression is regulated in a nuclear factor kappa B (NF-κB)-dependent manner, and its activation results in enhanced cell adhesion and migration (Tarnowski et al., 2010). In turn, knockdown of CXCR7 induced upregulation of CXCL12 mRNA expression and production in AML cell lines (Kim et al., 2015). However, in NOD/SCID mice, inhibition of CXCR7 impaired homing of both CD34^+^ cells and AML cell lines (Melo et al., 2018). Future research should elucidate the role of CXCR7 in LSC retention (Figure 2) and its potential as therapeutic strategy.

### Inflammatory Cytokines

G-CSF is produced in response to inflammation and infection. Initially described as anti-inflammatory modulator by inhibiting production of the main inflammatory mediators interleukin-1, tumor necrosis factor (TNF)-α and interferon (IFN)-γ, it was later recognized as pro-inflammatory factor by promoting trafficking of neutrophils (Hartung, 1998; Eyles et al., 2008). In HSC, G-CSF promotes granulocyte differentiation (Panopoulos and Watowich, 2008) and enforces their mobilization through proteolytic degradation of both CXCL12 and CXCR4 (Petit et al., 2002; Levesque et al., 2003). As mentioned above, plerixafor is approved by the FDA in combination with G-CSF for mobilizing HSC to the bloodstream. G-CSF treatment of NSG mice transplanted with CD34^+^ CD38^–^ AML patient bone marrow cells induced these cells to enter into cycle. In combination with cytarabine, G-CSF significantly increased apoptosis of CD34^+^ CD38^–^ cells *in vivo* and survival of secondary recipients (Saito et al., 2010). G-CSF has been tested therapeutically alone and in combination with plerixafor to treat R/R AML patients. In a phase I/II clinical trial (NCT01435343), R/R AML patients were treated with G-CSF plus fludarabine, cytarabine and idarubicin plus plerixafor (PLERIFLAG regimen). A high 49% achieved CR/CRi phase and from these, 61% underwent subsequent HSC allogenic transplantation. However, the median overall and disease-free survivals were low, 9.9 and 13 months, respectively (Martinez-Cuadron et al., 2018). In NOD/SCID mice, CXCR4 inhibitor AMD3465 in combination with G-CSF and protein kinase inhibitor sorafenib improved survival and resulted in reduction of leukemia cells in bone marrow and their absence in spleen or liver (Zeng et al., 2009). Sorafenib was tested for toxicity in a phase I clinical trial in combination with G-CSF and plerixafor in AML patients with FLT3-ITD mutation (NCT00943943). A remarkable mobilization effect was observed; 30-fold for WBC, 40-fold for AML blasts and > 100-fold in case of CD34^+^ CD38^–^ CD123^+^ LSC. Overall response rate was 62%, including 28% CR and CR with incomplete platelet recovery (Andreeff et al., 2014). Clinical results on mobilization were encouraging and should be further improved by more potent FLT3 inhibitors.

In addition to CXCL12, other soluble factors may influence cell adhesion, survival and chemotherapy resistance in AML, potentially inflammatory cytokines through activation of NF-κB pathway as it is the case in other types of cancer (Hoesel and Schmid, 2013). The glucocorticoid dexamethasone is currently in a phase III clinical trial in R/R AML in combination with intensive chemotherapy amsacrine-cytarabine or azacitidine (NCT03765541), and was reported to improve relapse incidence, disease-free survival, event-free survival, and overall survival compared to chemotherapy alone (Bertoli et al., 2018). Using a xenotransplantation model of chemotherapy resistance in NSG mice, the authors showed that dexamethasone treatment in AML patients may sensitize AML cells to chemotherapy-induced cell death in xenografts (Bertoli et al., 2018).

In fact, LSC but not healthy HSC or AML blasts display constitutive NF-κB activity through autocrine TNF-α secretion in several AML mouse models (Krause and Van Etten, 2007; Kagoya et al., 2014). Enhanced activation of NF-κB signaling expanded LSC and inhibition of the proteasome slowed down AML progression *in vivo*. Given that this pathway is activated in human CD34^+^CD38^–^ LSC but not in normal HSC, it stands out as a promising approach to target LSC (Kagoya et al., 2014). Several clinical phase I and II trials using proteasome inhibitors, like bortezomib or its second-generation counterpart ixazomib, combined with different chemotherapy regimens were conducted in AML patients with various degrees of success (NCT00383474, NCT00382954, NCT00666588, NCT02070458, NCT02030405, NCT00963495). Recently, preliminary results were published from a phase I trial with ixazomib in combination with MEC in patients with R/R AML (NCT02070458). The overall CR and CRi was 53%, with median overall survival for all patients of 4.5 months, and 11.1 months for patients achieving CR/CRi (Advani et al., 2019). Future randomized trial will address if outcome is improved by addition of ixazomib to MEC.

Acute IFN-α treatment *in vivo* promotes proliferation of dormant HSC and makes them sensitive to 5-fluoro-uracil, whereas chronic activation of the IFN-α pathway in HSC impairs their function (Essers et al., 2009). Clinical phase I to IV trials have used IFN-α for prevention of relapse in AML with the chromosomal translocation t (8; 21), not in remission after allogeneic HSC transplantation, aiming at promoting graft vs. leukemia effect (NCT02328755, NCT02027064). After the treatment, 35.7, 11.9, 7.1, and 21.5% patients achieved minimal residual disease negativity at 1, 2, 3, and more than 3 months, respectively. The 1-year probabilities of event-free survival, disease-free survival, and overall survival after treatment were also promising; 76.0, 92.4, and 92.5%, respectively (Mo et al., 2018). These results suggest that IFN-α may be effective to eliminate LSC, and the underlying mechanisms should be investigated. In parallel, IFN-γ inhibits the generation of myeloid progenitors and prevents lineage differentiation from HSC leading to aplastic anemia, without infiltration of activated T cells in the bone marrow (Lin et al., 2014). Integrin αvβ3 was recently connected to IFN-γ and reported to intensify IFNγ−dependent suppression of HSC (Umemoto et al., 2017). Future research should focus on the potential role of this pathway in LSC.

## Adhesion Molecules Anchoring LSC to the Stem Cell Niche and Their Clinical Application in AML

### Integrins: VLA-4

Integrins are transmembrane glycoproteins expressed in HSC and LSC (Levesque and Winkler, 2016; Gruszka et al., 2019; Windisch et al., 2019). As described above, CXCL12 activates the integrins LFA-1 and VLA-4 on CD34^+^ cells to bind their respective ligands in the niche, and treatment of these cells with antibodies to VLA-4 or VLA-5 hampered engraftment in NSG mice (Peled et al., 2000). One of the most prominent integrins involved in AML is VLA-4, which interacts with fibronectin, ICAM-2 and VCAM-1 in the niche (Gruszka et al., 2019; Figure 2). Expression of VLA-4 is necessary for migration of CD34^+^ AML cells beneath M210B4 marrow stromal cells *in vitro* (Burger et al., 2003), and it correlates with poor survival in AML patients (Matsunaga et al., 2003). Antibodies against VLA-4 in combination with cytarabine prolonged survival in a SCID model of U937 cell transplant and reduced detection of U937 cells by PCR in different tissues including bone marrow at day 62 after transplantation, compared to cytarabine alone. This indicates that adhesion of leukemic cells via VLA-4 contributes to disease and chemotherapy resistance (Matsunaga et al., 2003). This was further confirmed by the chemical compound AS101, which disrupts VLA-4 function, and combined with cytarabine, abrogated chemotherapy resistance and prolonged survival in SCID mice transplanted with AML cells from a patient expressing high VLA-4 (Layani-Bazar et al., 2014). The safety and efficacy of AS101 was planned to be tested in AML patients in a phase II clinical trial that is currently suspended given that the sponsor is focusing on different indications (NCT01010373).

Of note, in a sample of newly diagnosed adult AML patients (*n* = 98), VLA-4 expression was high in those with favorable or intermediate cytogenetic risk. Further, among the 72 non-promyelocytic leukemia patients analyzed who received cytarabine and anthracycline, high VLA-4 expression was associated with high probability of complete remission and prolonged relapse-free survival (Bae et al., 2015). In pediatric AML, the association of high VLA-4 expression with better outcomes was mainly seen in patients with standard-risk disease, and possibly low-risk, but not in high-risk (Walter et al., 2010). Future work should elucidate the role of VLA-4 on LSC in the context of mutational profiling, and its implications in the clinic.

### CD98

CD98 heavy chain (CD98hc, SLC3A2) is a membrane protein that participates in fibronectin matrix assembly through interaction with integrins to enable downstream signaling (Feral et al., 2007; Figure 2). CD98 is expressed on human CD34^+^ cells (Bajaj et al., 2016). While conditional deletion of CD98hc in mice had no major effect on hematopoiesis, wild-type HSC outperformed HSC lacking CD98hc when transplanted in the same recipients (Bajaj et al., 2016).

CD98 in primary AML cells is overexpressed in more than 90% of AML patients (Hayes et al., 2015), and its expression was 3-fold higher in AML CD34^+^ cells than healthy cells (Bajaj et al., 2016). In NOD/SCID mice injected subcutaneously with the AML cell lines HL−60, OCI−AML−3, and KG−1, the anti-CD98 monoclonal antibody IGN523 reduced tumor growth by eliciting strong antibody−dependent cellular cytotoxicity (Hayes et al., 2015). CD98 knockdown with small hairpin RNA reduced bone marrow engraftment in NSG mice transplanted with two independent primary AML patient samples, and the monoclonal antibody IGN523 impaired both initiation and progression of human AML cell engraftment in xenografts. The same antibody had no effect on the ability of normal human CD34^+^ cells to engraft *in vivo*, pinpointing CD98 as a potential therapeutic target in AML (Bajaj et al., 2016). IGN523 was tested for safety, pharmacokinetics, and pharmacodynamics in a phase I clinical trial in R/R AML patients (NCT02040506). CD98 occupancy at higher doses was high, and there were evidence of transient anti-leukemic effects like reduction in blast count in 3 out of 19 patients. No complete or partial responses were observed, so its efficacy will be tested in combination with chemotherapy (Bixby et al., 2015).

### CD44 and Its Sialofucosylated Glycoform Hematopoietic Cell E-/L-Selectin Ligand (HCELL)

CD44 is a type I transmembrane protein that serves as adhesion molecule expressed by hematopoietic cells and at a higher level by stromal cells, whereas HCELL is expressed exclusively on healthy and AML hematopoietic cells, including HSC and LSC (Dimitroff et al., 2000; Peterson et al., 2007; Will and Steidl, 2010; Levesque and Winkler, 2016; Gruszka et al., 2019). When sialofucosylated as HCELL, CD44 binds to L-selectin and E-selectin, and CD44 is the receptor of other extracellular ligands like osteopontin, laminin, fibronectin, and hyaluronan (Zoller, 2015; Levesque and Winkler, 2016; Gruszka et al., 2019). CD44 and hialuronan cooperate with CXCL12 in human CD34^+^ progenitor cell homing to the bone marrow (Avigdor et al., 2004; Figure 2).

CD44 variant expression generated by alternative splicing shows increased complexity in human AML blasts than in healthy cells and CD34^+^ progenitors (Bendall et al., 2000). CD44v6 isoform expressing exon 6 confers additional properties like cooperation with various tyrosine kinase receptors, is the most abundant isoform expressed in AML and correlates with poor survival in AML patients (Legras et al., 1998; Nicolis di Robilant et al., 2013). AML1-ETO and its splicing variant containing the exon 9a AML1-ETO9a bind to CD44 promoter and increase its expression (Peterson et al., 2007). However, in an AML1-ETO9a^+^ mouse model, CD44 expression in LSC was regulated in a tissue-dependent manner; increased in spleen and blood, and decreased in bone marrow. Expression of CD44 in AML1-ETO9a^+^ LSC in the periphery could promote extramedullary leukemia potential, which has been reported in some t (8; 21) patients and may be associated to their poor prognosis. CD44 is also expressed in low levels in the BM of t (8; 21) AML patients (Peterson et al., 2007). Using a HOXA10^+^ transgenic model, other authors showed that high levels of CD44 on leukemic cells are essential for AML relapse after removal of the initial HOXA10 transforming event (Quéré et al., 2011). Administration of activating anti-CD44 monoclonal antibody H90 into NOD/SCID mice transplanted with human AML cells markedly reduced leukemic engraftment and induced blast differentiation (Jin et al., 2006). Eradication of LSC was confirmed by serial transplantations *in vivo*, and it was mediated by inhibition of the homing capacity of LSC into bone marrow or spleen (Jin et al., 2006).

These promising results are currently undergoing translation into the clinic. A very exciting phase I/II clinical trial is currently recruiting patients to evaluate the safety and antitumor activity of autologous CD44v6 chimeric antigen receptor (CAR) T-cells in R/R AML patients (NCT04097301). Briefly, patient autologous T-cells will be expanded *in vitro* in large numbers and genetically engineered to express the CAR CD44v6ΔNL gene. As a safety measure, the MLM-CAR44.1 T-cells will be additionally modified to express the suicide gene HSV-TK Mut2, which will be activated in case of toxicity through administration of ganciclovir.

### E-Selectin

Selectins play an important role in the interaction between HSC and LSC and the vascular niche (Winkler et al., 2012, 2014). E-selectin is expressed by bone marrow endothelial cells, and its deletion *in vivo* increased HSC quiescence and self-renewal, and enhanced HSC survival 3-fold to 6-fold after treatment of mice with chemotherapy or irradiation (Winkler et al., 2012). These effects were not mediated by the canonical E-selectin ligands P-selectin glycoprotein ligand-1 (PSGL-1, or CD162) or HCELL (Winkler et al., 2012; Figure 2).

Soluble E-selectin (sCD62E), together with sVCAM-1, are increased in serum of newly diagnosed AML patients, suggesting endothelial activation in AML (Kupsa et al., 2016, 2017). Conversely, AML blasts and LSC express E-selectin ligands, including PSGL-1, cutaneous lymphocyte antigen (CLA) and CD44 (Kappelmayer et al., 2001; Chien et al., 2013). In an AML mouse model generated by retroviral transduction of MLL-AF9 into HSC, LSC adhesion through E-selectin in the vascular niche protected LSC from chemotherapy (Winkler et al., 2014). Injection of E-selectin antagonist GMI-1271 mobilized AML blasts into the bloodstream, and deletion of E-selectin in primary recipients of AML cells increased LSC sensitivity to cytarabine treatment, assayed by limiting-dilution transplantation assays in wild-type recipients (Winkler et al., 2014). GMI-1271 showed similar positive results in AML xenografts in NSG mice, where it reduced leukemia burden in combination with daunorubicin and cytarabine (Chien et al., 2013).

This encouraged the evaluation of GMI-1271 (Uproleselan) in a phase I/II clinical trial in combination with MEC in R/R AML patients, or added to cytarabine and idarubicin in newly diagnosed elderly AML patients (NCT02306291). In R/R patients, CR/CRi rate obtained was 41% at the recommended dose of uproleselan, median overall survival was 8.8 months, remission duration was 9.1 months and 1-year overall survival was 35%. High E-selectin-ligand expression, studied by percentage of binding to E-selectin-Fc chimeric protein and HECA452, in both AML blasts and LSC was associated with improved remission and survival with uproleselan treatment in those patients. In newly diagnosed older patients, CR/CRi was 72%, median overall survival was 12.6 months, remission duration 10.4 months and 1-year overall survival was 52%. In spite of better outcomes, in these patients E-selectin-ligand on LSC was similar and low between responders and non-responders. However, the median overall survival for patients with more than 10% of E-selectin-ligand on LSC was 10.5 months, vs. not reached for those with less 10% (DeAngelo et al., 2018). Future studies should aim at understanding these differences, but the results are promising. Two randomized clinical trials are currently recruiting patients; a phase III trial will evaluate uproleselan administered with chemotherapy vs. chemotherapy alone in R/R AML patients (NCT03616470), and a phase II/III study will test conventional chemotherapy with or without uproleselan in older adults (NCT03701308).

### Cadherins

Cadherins are glycoproteins whose extracellular domains interact to form cell–cell adhesions (Perez and Nelson, 2004). N- and E-cadherins are expressed by both stromal cells and subsets of HSC (Turel and Rao, 1998; Puch et al., 2001; Frederik et al., 2010; Hosokawa et al., 2010; Figure 2). N-cadherin conditional knock-out mice showed no defects in HSC (Kiel et al., 2009), while its overexpression in HSC promoted quiescence and preserved their function in serial transplantations (Hosokawa et al., 2010). Increased numbers of N-cadherin^+^ osteoblastic cells correlated with higher numbers of HSC (Zhang et al., 2003), but others showed that N-cadherin from osteoblasts is not required for HSC maintenance (Bromberg et al., 2012; Greenbaum et al., 2012). In AML, N-cadherin^+^ LSC, identified as CD34^+^CD38^–^CD123^+^, induced disease in primary and secondary xenograft recipients, slightly more efficiently than N-cadherin^–^ LSC (Qiu et al., 2014). In addition, N-cadherin was highly expressed in bone marrow mononuclear cells from AML patients with t (8; 21) translocation (Zhi et al., 2016).

Data on HSC expression of E-cadherin are scarce, whereas low expression of E-cadherin was reported in AML blasts compared to CD34^+^ and associated to differentiation blockage along HL-60 cells (Melki et al., 2000; Ewerth et al., 2016). In the same line, a recent study showed that small interfering RNA downregulation of E−cadherin reduced adhesion of THP-1 cells to UE6E7T−3 mesenchymal cells and enhanced the effect of cytarabine (Nishioka et al., 2017). Future studies should evaluate further the role of cadherins in AML as well as their therapeutic potential.

### Eph Receptors

Eph receptors are transmembrane tyrosine kinase receptors that mediate cell–cell communication by interacting with their ligands ephrins on neighboring cells. High gene expression levels of EphA1, ephrin-A2, ephrin-A3, and ephrin-B2 were reported in human bone marrow CD34^+^ HSC (Ivanova et al., 2002; Steidl et al., 2004; Nguyen et al., 2016), and protein analyses detected EPHA2 and EPHB2 in human mobilized CD34^+^ HSC (Lazarova et al., 2006). Their function is yet unclear. Eph gene expression was low in the bone marrow of AML patients compared to controls. Further, low EphA4 levels were associated with higher leukocytes and blasts, and FLT3-ITD mutation. Conversely, low EphB2 expression correlated inversely with CR rate and overall survival (Wrobel et al., 2014).

Interestingly, EphA3 is not expressed by normal hematopoietic cells but it is present on leukemic blasts in a high percentage of AML and MDS patients. In the NUP98-HOXD13 (NHD13) mouse model of MDS, EphA3 was expressed on LSC that home to the bone marrow and co-localize with CXCL12 (Slape, 2014; Figure 2). EPHA3 was also expressed on a subset of immunophenotypically defined LSC, CD34^+^ CD38^–^ CD123^+^, in AML patients, and the high-affinity recombinant antibody to EPHA3, KB004, reduced the numbers of long-term culture initiating cells *ex vivo* (Palath et al., 2010). A phase I/II clinical trial was conducted using KB004 in patients with advanced hematological malignancies, including AML (NCT01211691), and showed that it was well tolerated and clinically active (Swords et al., 2016).

EPHB4 is highly expressed in ∼30% of AML samples. In xenografts of NSG mice transplanted with EPHB4^+^ primary AML cells, targeting EPHB4 using the monoclonal antibody MAb131 markedly improved survival, particularly in combination with cytarabine (Merchant et al., 2017). Future work should evaluate further the clinical value of targeting EPH in human AML.

### GPR56

GPR56 (ADGRG1) is a member of the adhesion G protein-coupled receptors (adhesion GPCRs) that can partner with collagen III and heparin, among others (Huang and Lin, 2018; Figure 2). In mice, GPR56 is highly and selectively expressed by HSC, but its deletion showed little effect on steady-state or regenerative hematopoiesis, other than mobilization of HSC from bone marrow to periphery *in vivo* and reduced adhesion to different substrates assessed *ex vivo* (Saito et al., 2013; Rao et al., 2015).

In human AML, GPR56 mRNA level was higher in intermediate- and high-risk samples than in the low-risk group (Saito et al., 2013; Pabst et al., 2016). GPR56 was identified in a family of LSC-related genes that correlated with worse prognosis in patients (Eppert et al., 2011). It was subsequently described as a marker of high reconstitution potential, within both CD34^–^ and CD34^+^ subsets (Pabst et al., 2016), and reported as higher expressed in the CD34^+^ CD38^–^ AML compartment, enriched in LSC (Daga et al., 2019). In fact, high expression of GPR56 in CD34^+^ CD38^–^ AML correlated with LSC gene expression signature and reduced survival in patients receiving intensive chemotherapy (Daga et al., 2019).

The ecotropic viral integration site-1 (EVI1) transcription factor promotes stemness and it is a marker of poor prognosis for chemotherapy-resistant AML (Eppert et al., 2011). GPR56 mRNA was highly expressed in EVI1^high^ AML patient samples compared to EVI1^low^ counterparts (Saito et al., 2013). Conversely, interference with the binding of EVI1 to the GPR56 promoter through GPR56-specific pyrrole-imidazole polyamides treatment of immunodeficient Balb/c-RJ mice subcutaneously transplanted with the EVI1^high^ UCSD/AML1 cell lines inhibited tumor growth and improved survival (Saha et al., 2018). Future work should evaluate the clinical potential of this promising strategy.

### Junctional Adhesion Molecule (JAM)-C

JAM-C is expressed on HSC and binds to JAM-B expressed on stromal cells in both humans and mice (Figure 2), and the anti−Jam−C monoclonal antibody 13H33 inhibited HSC reconstitution and homing, and induced HSC mobilization in a Jam−b dependent manner in mice (Arcangeli et al., 2014). Recently, JAM-C–expressing cell frequency in peripheral blood was identified as prognostic marker for poor disease outcome in AML patients (De Grandis et al., 2017). Limiting dilution assays in NSG mice using CD34^+^ CD38^low^ CD123^+^ CD41^–^ JAM-C^+^ or JAM-C^–^ cells isolated from AML patients showed that JAM-C^+^ cells were 9.5-fold enriched in LSC compared with JAM-C^–^ cells. Another recent study showed JAM-C expression on bulk leukemic cells and inversed correlation between CD34 and JAM-C expression, with still reduced overall survival in JAM-C^+^ patients (von Bonin et al., 2018).

JAM-C was related to overactivation of Src family kinase (SFK) in LSC (De Grandis et al., 2017), pathway previously described as constitutively active in many cases in AML patients and involved in signal transduction from a variety of receptors including FLT3 (Robinson et al., 2005; Dos Santos et al., 2008). SFK blockade is currently in phase II and III clinical trials combining dasatinib (sprycel) with cytarabine, daunorubicin and idarubicin in adult patients with newly diagnosed core-binding factor (CBF)-AML (NCT01238211, NCT02013648). The rationale to use dasatinib against CBF-AML grounded on the frequently high KIT receptor tyrosine kinase expression and/or presence of KIT mutations in CBF-AML, as well as dasatinib efficacy against both wild-type and mutant KIT (Paschka et al., 2018).

## Discussion

AML is a devastating disease, whose treatment has changed little for decades and relies on the use of aggressive chemotherapy. Chemotherapy is effective in reducing AML bulk and extending survival short-term, but promotes relapse in the long-run by selection of chemoresistant LSC. These cells may not only resist chemotherapy, but diversify to drive progression to more aggressive forms of AML (Vetrie et al., 2020). It is then clear that any potential therapy for AML should ultimately aim at LSC eradication.

*In vivo* models suggest that LSC outcompete HSC for the same bone marrow niche, and transform it to their advantage. The bone marrow niche helps LSC stay dormant and protected from chemotherapy. Thus, theoretically, LSC release from the niche will force them into cycle and make them sensitive to chemotherapy. Further understanding of the similarities and differences between HSC and LSC, and their interaction with their microenvironment are required to help open new avenues for potential therapies against AML. The role of CXCL12/CXCR7, cytokines like IFN, cadherins, Eph receptors/ephrins, and JAM-C/JAM-B, among others, require further thorough characterization in this context.

Preclinical data show that targeting LSC anchoring to the niche has therapeutic potential in AML. A promising target recently pinpointed by *in vivo* research that should be evaluated in the clinic is GPR56. A good number of preliminary phase I/II clinical trials are studying or have studied the safety and efficacy of a variety of strategies targeting homing and adhesion, alone or in combination with chemotherapy; i.e., plerixafor alone or combined with G-CSF, ulocuplumab, proteasome inhibitors, AS101 (currently suspended), CD44v6 CAR T cells and IGN525. Overall, treatments were well tolerated in patients. However, these trials often study low numbers of patients, sometimes in non-randomized cohorts where scalating agent doses are tested, making results on efficacy difficult to interpret and generally low.

Some other strategies are already under phase III clinical trials or will soon follow up into this phase due to promissing response rates. Phase III studies are usually randomized and evaluate a large number of patients over a long period of time, providing grounds for robust conclusions. This is the case of CXCR4 antagonist peptides, ODSH, dexamethasone and uproleselan. Exciting times are ahead of us, with potential shift in the focus of AML patient management to LSC-directed therapies and prospect of improved cure rates.

However, it is necessary to consider that AML is a heterogeneous and complex disease (Coombs et al., 2016), and clonal evolution complexity has just started to be unraveled at the single-cell level (Chen et al., 2019). We cannot expect a simple cure. For the same reasons, it is difficult to predict which of the strategies discussed in the present work will be more successful. Thorough immunophenotypic LSC characterization may help select patients with best chances of success for specific targeted novel therapies. Immunophenotyping should map further than CD34^+^ CD38^–^, as this is a cell fraction only enriched and not fully overlapped with LSC. In fact, functional LSC have been detected in CD34^+^ CD38^–^ and CD34^+^ CD38^+^, but also CD34^–^ compartments (Ishikawa et al., 2007; Eppert et al., 2011; Sarry et al., 2011). When testing the efficiency of these therapies, eradication of LSC should be confirmed functionally by *in vivo* xenografts to at least secondary and best tertiary recipients, as quiescent LSC may arise only in these (Hope et al., 2004). Limiting-dilution xenotransplants should be avoided because they may underestimate the numbers of LSC due to inefficient homing and/or engraftment of single LSC (Somervaille and Cleary, 2006). Serial transplantations are not practical in the clinic as they are time-consuming, and also limited given that they do not fully mimic the settings of the human disease, including human LSC niche nuances. To overcome these limitations, evaluation of LSC should combine *in vivo* functional studies with genetic studies. Recent developments in this field make it now possible to analyze the stemness signatures and mutational profiles at the single-cell level. In the era of precision medicine, the future of AML treatment should lean toward thorough selection of patients for personalized therapies using combinations of mutation-targeted agents, non-mutation-targeted novel agents like those under development described in this work and/or targeted delivery of chemotherapeutic agents, ultimately aiming at LSC eradication with reduced side-effect burden.

## Author Contributions

All authors listed have made direct and intellectual contribution to the work, and approved it for publication.

## Conflict of Interest

The authors declare that the research was conducted in the absence of any commercial or financial relationships that could be construed as a potential conflict of interest.
